# HDAC9 Is Preferentially Expressed in Dedifferentiated Hepatocellular Carcinoma Cells and Is Involved in an Anchorage-Independent Growth

**DOI:** 10.3390/cancers12102734

**Published:** 2020-09-23

**Authors:** Keita Kanki, Ryota Watanabe, Le Nguyen Thai, Chun-Hao Zhao, Kyoko Naito

**Affiliations:** Department of Biomedical Engineering, Faculty of Engineering, Okayama University of Science, 1-1 Ridai-cho, Kita-ku, Okayama 700-0005, Japan; linsecome@icloud.com (R.W.); t19mm01nt@ous.jp (L.N.T.); t20mm01zc@ous.jp (C.-H.Z.); t20mm02nk@ous.jp (K.N.)

**Keywords:** histone deacetylase, stemness, aldehyde dehydrogenase, hepatocellular carcinoma

## Abstract

**Simple Summary:**

Histone deacetylases (HDACs) are known to play a role in malignant transformation of cancer cells, however, the critical HDAC responsible for the dedifferentiation of hepatocellular carcinoma (HCC) cells remains unclear. The aim of our study was to identify the HDAC related to the dedifferentiation of HCC. We confirmed preferential expression of HDAC9, a class II HDAC, in undifferentiated hepatoma cells and a positive correlation of gene expression between HDAC9 and dedifferentiation markers by database analysis of HCC patients. Genetic and pharmacological inhibition of HDAC9 showed decreased cell proliferation and sphere-forming activity, which indicates an ability of anchorage-independent cell growth and self-renewal. HDAC9 suppression showed significant down-regulation of aldehyde dehydrogenase 1A3 (ALDH1A3), a stemness-related gene reported in several malignancies including HCC. We also confirmed that ALDH activity is required for the anchorage-independent cell growth of undifferentiated HCC cells. Inhibition of HDAC9 may be a therapeutic strategy for targeting dedifferentiated HCC cells with stemness features.

**Abstract:**

Aberrant activation of histone deacetylases (HDACs) is one of the causes of tumor cell transformation in many types of cancer, however, the critical HDAC responsible for the malignant transformation remain unclear. To identify the HDAC related to the dedifferentiation of hepatocellular carcinoma (HCC) cells, we investigated the expression profile of HDACs in differentiated and undifferentiated hepatoma cells. We found that HDAC9, a member of the class II HDAC, is preferentially expressed in undifferentiated HCC cells. Analysis of 373 HCC patients in The Cancer Genome Atlas (TCGA) database revealed that the expression of HDAC9 mRNA positively correlated with the markers of mesenchymal phenotype and stemness, and conversely, negatively correlated with hepatic differentiation markers. HDAC9 was transcriptionally upregulated in epithelial–mesenchymal transition (EMT)-induced HCC cells treated with TGF-β. Genetic and pharmacological inhibition of HDAC9 in undifferentiated HCC cells showed decreased sphere-forming activity, which indicates an ability of anchorage-independent cell growth and self-renewal. We also showed that aldehyde dehydrogenase 1A3 (ALDH1A3) was downregulated in HDAC9-suppressing cells, and ALDH inhibitor disulfiram significantly decreased the sphere formation of undifferentiated HCC cells. Together, our data provide useful information for the development of HDAC9-specific inhibitors for the treatment of HCC progression.

## 1. Introduction

Histone acetylation and deacetylation are known epigenetic modifications, which can play a role in a gene regulation. Normally, histone acetyltransferase (HAT)-mediated histone acetylation results in a more relaxed chromatin structure that allows the transcription complexes to interact and activate the gene transcription. Inversely, deacetylation of histones is catalyzed by histone deacetylases (HDACs), and functions as a repressive mechanism of gene transcription by promoting chromatin condensation [1]. As a consequence, aberrant activation of HDACs is considered to be involved in a process of carcinogenesis and malignant progression of tumor through silencing the tumor-suppressor genes.

HDACs are categorized into four classes; class I (HDAC1, 2, 3, and 8), class II (HDAC4, 5, 6, 7, 9 and 10), class III (Sirtuin1, 2, 3, 4, 5, 6 and 7), and class IV (HDAC11) [2]. Classically, class I HDACs have been mainly investigated and reported to be involved in the pathogenesis and poor prognosis of patients in many types of cancers. Small molecules that block the enzymatic activity of HDACs (HDAC inhibitors), such as tricostatin A, valproic acid, romidepsin, and vorinostat, have been approved or are under investigation for the promising cancer treatment. However, these agents have possible side effects caused by inhibition of a broad range of HDACs [3]. Moreover, which HDAC is responsible for the malignant behavior of cancer cells depends on the type of tissues. Therefore, it is important to identify which HDAC plays a pivotal role in the malignant transformation of cancer cells of the target organ.

Hepatocellular carcinoma (HCC), the most frequently diagnosed primary liver cancer, is an aggressive disease caused by chronic hepatitis B or C virus infection, excessive alcohol intake, non-alcoholic steatohepatitis, or exposure to aflatoxin B [4]. The prognosis for patients with advanced stage HCC is poor because of its high rates of recurrence and intrahepatic metastasis. Consequently, there is an essential requirement for the development of an improved therapeutic strategy that aims to inhibit HCC progression. The malignant transformation of tumor cells is related to dedifferentiation of the cells [5]. In addition to the primary dedifferentiation events such as increased proliferation, enhanced glycolytic activity, and a loss of cellular specific functions, subsequent genetic and epigenetic alteration often cause a loss of cell–cell adhesion, increased cell motility, and anchorage-independent cell proliferation, which are the crucial malignant phenotypes that trigger invasiveness and metastasis [6]. In HCC, class I HDAC1 and HDAC2 were mainly reported to be associated with poor prognosis [7,8], but inhibition of class II HDAC, HDAC4 and HDAC5 has also been reported to have significant anti-cancer effects [9,10]. A number of HDAC inhibitors have been shown to be effective in preventing the growth of HCC as well as the other types of cancer, however, the pivotal HDAC responsible for the dedifferentiation of HCC cells remains unclear.

In this study, we analyzed the gene expression of a panel of HDACs and found that expression of HDAC9, one of the class II HDAC, is positively correlated with a dedifferentiated phenotype of HCC cells. Functional analysis using genetic and pharmacological inhibition revealed that its expression is required for the anchorage-independent cell growth in undifferentiated HCC cells. We also found a possible link between HDAC9 and aldehyde dehydrogenase (ALDH) and showed a correlation of ALDH activity to the stemness feature. Our data provide useful information for preventing malignant progression of HCC by targeting specific HDACs.

## 2. Results

### 2.1. HDAC9 Gene Expression Correlates with a Dedifferentiated Phenotype of HCC Cells

**Figure 1:**
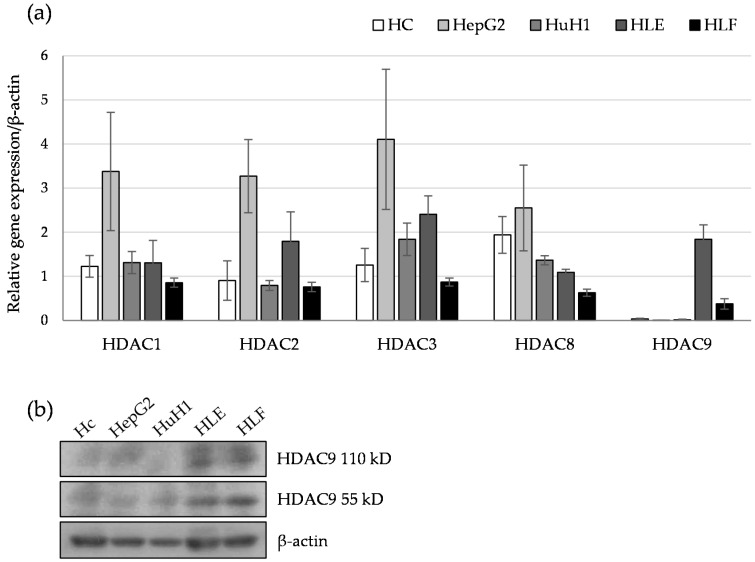
Differential expression of histone deacetylases (HDACs) in differentiated and undifferentiated hepatocellular carcinoma (HCC) cell lines. (**a**) Relative gene expression levels of HDAC1, HDAC2, HDAC3, HDAC8, and HDAC9 compared to a house keeping gene (β-actin) were determined in two differentiated (HepG2 and HuH1) and two undifferentiated (HLE and HLF) type HCC cell lines by quantitative PCR analysis (*n* = 3). The human hepatic cell line (Hc) was used as a non-transformed liver cell. (**b**) Western blot analysis of HDAC9 and β-actin as a protein loading control. Uncropped western blot figures in Figure S4.

To determine which HDAC is involved in the dedifferentiation of HCC cells, a gene expression analysis of class I, class II, and class IV HDAC genes was performed on a panel of hepatoma cells including two differentiated (HepG2 and HuH1) and two undifferentiated (HLE and HLF) types of HCC cells. The human hepatic cell line Hc was used as a non-transformed liver cell. At first, we performed RT-PCR analysis of HDAC genes and obtained positive amplification with the primers for HDAC1, HDAC2, HDAC3, HDAC8, and HDAC9 genes, while the other HDACs (HDAC4, HDAC5, HDAC6, HDAC7, HDAC10, and HDAC11) were hardly expressed [11]. Quantitative PCR experiments revealed that a measurable expression of class I HDAC genes were observed in all cell lines examined (Figure 1a). In contrast, the expression of HDAC9 was preferentially expressed in undifferentiated HCC cells, and not in differentiated HCC cells and normal hepatic cells (Hc). Western blot revealed the expression of two putative isoforms of HDAC9 in undifferentiated HCC cells (Figure 1b). Next, we focused on HDAC9 gene expression and searched the genes that show positive or negative correlation with HDAC9 by screening The Cancer Genome Atlas (TCGA) database. Analysis of 373 liver cancer datasets revealed that the expression profile of HDAC9 positively correlated with those of stemness-related genes, mesenchymal genes, and cell-cycle regulators (Table 1). On the other hand, negatively-correlated genes include hepatic differentiation markers, such as HNF1A, HNF4A, and FOXA2. These data suggest that, among HDACs, HDAC9 may be related to the dedifferentiated status of HCC cells.

### 2.2. HDAC9 is Induced during the Dedifferentiation Process of EMT Induced by TGF-β

**Figure 2:**
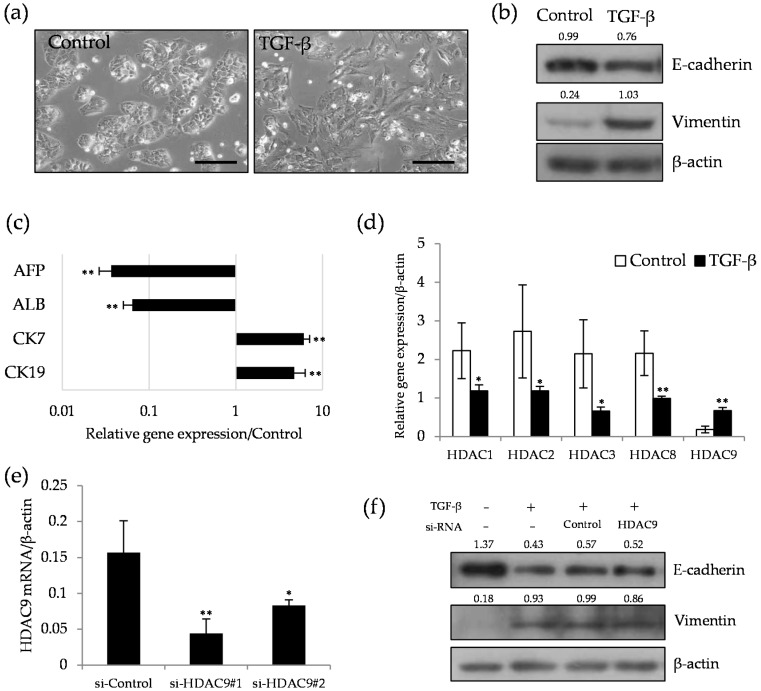
Selective induction of HDAC9 in transforming growth factor-β (TGF-β)-induced epithelial–mesenchymal transition (EMT) in differentiated HCC cells. (**a**) Microscopic observation of HuH1 cells treated with control vehicle (**left**) and 10 ng/mL of TGF-β (**right**) for 48 h. Note the morphological change from epithelial to mesenchymal cell shape by TGF-β stimulation. Scale bar, 50 μm. (**b**) Western blot analysis of E-cadherin, vimentin, and β-actin as a protein loading control. Relative protein expression levels of β-actin were calculated from the band intensity with ImageJ software (NIH) and described above each band. Uncropped western blot figures in Figure S4. (**c**) Quantitative PCR analysis of hepatic (alpha fetoprotein; AFP and albumin; ALB) and hepatic progenitor (keratin 7; CK7 and keratin 19; CK19) markers in TGF-β-treated cells. The values are expressed as relative expression compared to vehicle control group. ** *p* < 0.01 vs. the vehicle control. (**d**) Relative gene expression of HDACs to β-actin in HuH1 cells treated with control vehicle (open bar) and TGF-β (closed bar) for 48 hrs. * *p* < 0.05, ** *p* < 0.01 vs. the vehicle control. (**e**) HDAC9 transcript level in HLF cells transduced with si-RNA for negative control (si-control) and HDAC9 (si-HDAC9#1 and #2). * *p* < 0.05, ** *p* < 0.01 vs. si-control. (**f**) Western blotting of E-cadherin, vimentin in the cells treated with TGF-β and si-RNAs. Cells were pretreated with mock (lipofectamine), si-Control, and si-HDAC9#1 for 24 h and subsequently stimulated with TGF-β to induce EMT for 48 h. Cells without any treatment served as non-treated control. Quantitative PCR analysis was performed at *n* = 4. Western blot was performed at least three times, and the representative blot was shown. Uncropped western blot figures in Figure S4.

Next, we investigated the expression profile of HDAC genes in the cells that received EMT induction, a dedifferentiation phenomenon of epithelial tumor cells, by TGF-β stimulation. Differentiated type hepatoma HuH1 cells showed marked morphological changes from epithelial to mesenchymal type after 48 h of TGF-β treatment (Figure 2a). Consistent with this change, decreased E-cadherin and increased vimentin expression were observed by Western blot analysis (Figure 2b). Quantitative PCR showed that hepatic markers AFP and Albumin were dramatically decreased by EMT induction, and in contrast, hepatic progenitor markers CK7 and CK19 were significantly up-regulated (Figure 2c). In this dedifferentiation process, HDAC9 was the only up-regulated gene while class I HDAC genes were uniformly down-regulated (Figure 2d). We examined whether HDAC9 knockdown by siRNA affected the phenotypic change of TGF-β-induced EMT. Although si-HDAC9#1 transduction significantly down-regulated the expression of HDAC9 mRNA, protein expression of E-cadherin and vimentin was not altered between si-control and si-HDAC9 groups (Figure 2e,f). Similarly, wound-healing cell migration assay, a functional parameter for mesenchymal phenotype, showed no significant difference between si-control and si-HDAC9 groups (Figure S1). These observations suggest that HDAC9 may play a role in the dedifferentiated, mesenchymal phenotype, but has no impact on the EMT by TGF-β stimulation.

### 2.3. HDAC9 is Involved in the Sphere Formation Capacity of Undifferentiated HCC Cells

**Figure 3:**
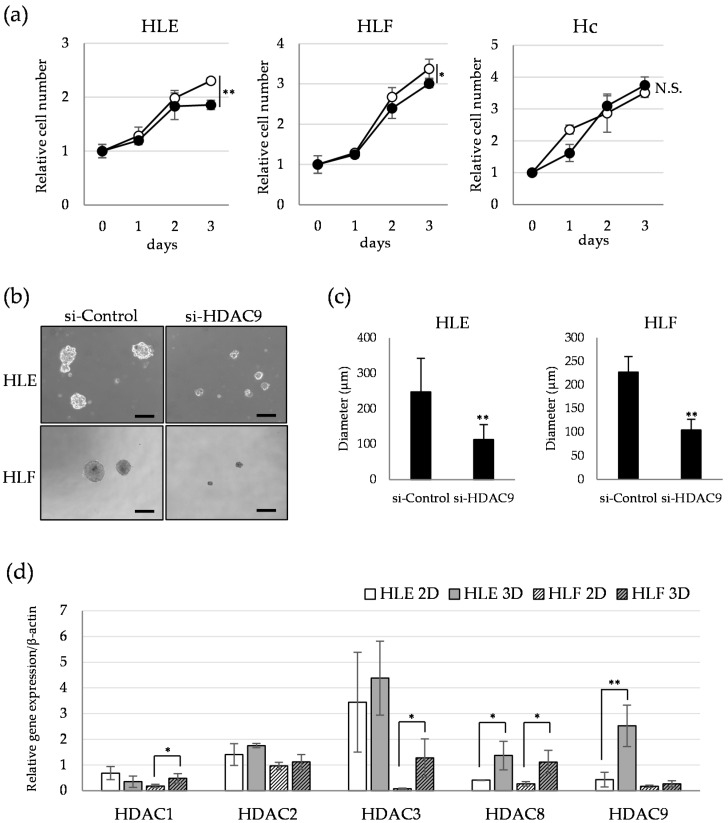
Involvement of HDAC9 in the sphere formation capacity of undifferentiated HCC cells. (**a**) Cell proliferation assays of HLE (**left**), HLF (**center**), and Hc (**right**) cells treated with si-Control (open circle) and si-HDAC9 (closed circle) for 3 days. Cell viability was determined by water-soluble tetrazolium (WST) assay and expressed as the relative amount of viable cells compared to day 0. * *p* < 0.05, ** *p* < 0.01 vs. si-Control group, N.S.: not significant, *n* = 3 (**b**) Morphological appearance of spheres formed in the culture medium containing 10 pmol/mL si-Control (**left**) and si-HDAC9 (**right**). HLE (**upper**) and HLF (**lower**) cells were cultured in an ultra-low attachment culture plate for 7 days and photographed. Scale bar = 200 µm. (**c**) The diameter of the sphere was measured in ten representative spheres of each group. ** *p* < 0.01 vs. si-control. (**d**) Relative gene expression levels of HDAC1, HDAC2, HDAC3, HDAC8, and HDAC9 compared to a house keeping gene (β-actin) were measured in 2D-(monolayer) and 3D-(sphere) cultured HLE and HLF cells by quantitative PCR analysis (*n* = 4). * *p* < 0.05, ** *p* < 0.01 vs. 2D-cultured group. Quantitative PCR analysis was performed at *n* = 4.

To investigate the impact of HDAC9 knockdown on cell growth, we assayed the proliferation activity under the conditions of adhesive and non-adhesive culture. HDAC9 knockdown by si-RNA significantly decreased the proliferation of HLE and HLF cells in monolayer culture at day 3, and not in those of Hc cells (Figure 3a). Next, we evaluated the effect of HDAC9 knockdown on the sphere formation activity in suspension culture by using a low-attachment culture surface. Undifferentiated HCC cells transfected with si-HDAC9 showed decreased sphere-forming activity, which indicates the ability of anchorage-independent growth (Figure 3b,c). We compared the profile of HDAC gene expression in 2D and 3D cultured HCC cells. Quantitative PCR revealed that a significant increase in HDAC9, HDAC8, and HDAC3 was observed in 3D-cultured HCC cells compared to those of 2D-cultured cells (Figure 3d). Sensitivity to cell death was examined by exposing control and HDAC9-surpressed cells to various concentrations of mitomycin C and sorafenib, two drugs with different anti-cancer mechanisms. Cell viability assay revealed that HDAC9 knockdown by si-RNA barely affected the induction of cell death by these drugs (Figure S2). These results suggest that undifferentiated HCC cells with sphere-forming capacity show higher HDAC9 expression than a monolayer-cultured population, and its expression is required for their anchorage-independent growth.

**Figure 4:**
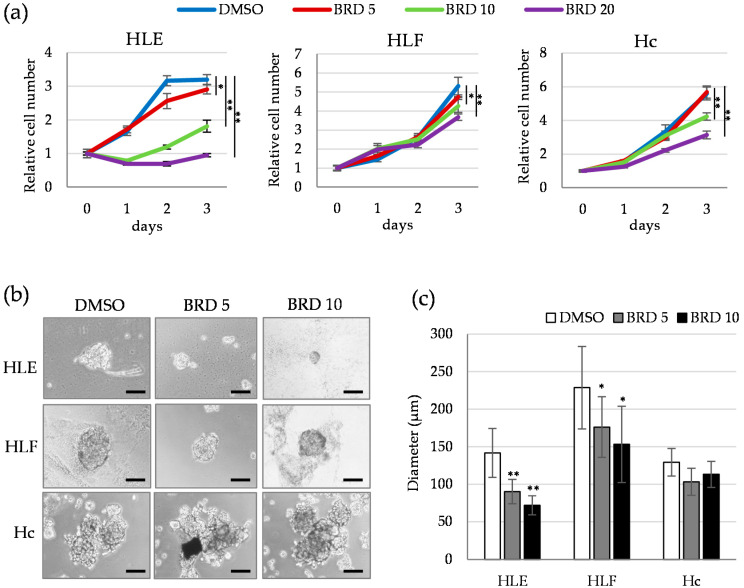
Small molecule-mediated inhibition of HDAC9 decreased the cell growth of monolayer- and 3D-cultured HCC cells. (**a**) Cell proliferation assays of HLE (**left**), HLF (**center**), and Hc (**right**) cells treated with dimethyl sulfoxide (DMSO) (vehicle control) and BRD4354 (5, 10, and 20 μM) for 3 days. Cell viability was determined by WST assay and expressed as the relative amount of viable cells compared to day 0. * *p* < 0.05, ** *p* < 0.01 vs. DMSO group. (**b**) Morphological appearance of spheres formed in the culture medium containing DMSO and BRD4354 (5 and 10 μM). Cells were cultured in an ultra-low attachment culture plate for 7 days and photographed. Scale bar = 200 µm. (**c**) The diameter of the sphere was measured in ten representative spheres of each group. * *p* < 0.05, ** *p* < 0.01 vs. DMSO group.

We next investigated the effect of small molecule-mediated inhibition of HDAC9 on the proliferation and sphere-forming activity. BRD4354, which preferentially inhibits activity of HDAC5 and 9, showed potent anti-proliferative activity in HLE cells, and to a lesser extent in HLF and Hc cells (Figure 4a). Sphere formation was significantly and dose-dependently inhibited by 5 and 10 μM of BRD4354 in HDAC9-positive HLE and HLF cells, and not in HDAC9-negative Hc cells (Figure 4b,c). Consistent with the result of Figure S1, BRD4354 barely inhibited the migration of HLE, HLF, and Hc cells (Figure S3). Together, genetic and pharmacological inhibition of HDAC9 revealed that HDAC9 plays an important role in cell proliferation and anchorage-independent cell growth in undifferentiated HCC cells.

### 2.4. Involvement of ALDH Activity in the Sphere Formation Capacity of HDAC9-Expressing Undifferentiated HCC Cells

**Figure 5:**
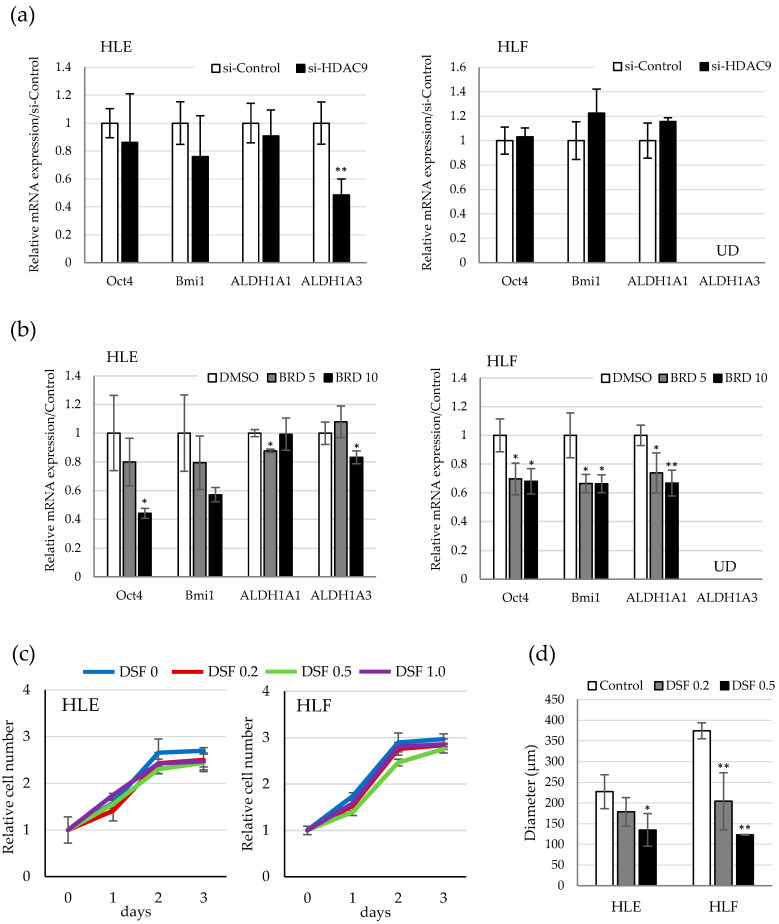
Involvement of aldehyde dehydrogenase (ALDH) activity in the sphere formation capacity of HDAC9-expressing undifferentiated HCC cells (**a**) Quantitative PCR of stemness-related genes Oct4, Bmi1, ALDH1A1, and ALDH1A3 in HLE (**left**) and HLF (**right**) cells treated with si-Control and si-HDAC9. The graphs are expressed as the relative expression to those of the si-Control group. UD; undetectable, ** *p* < 0.01 vs. si-Control. (**b**) Quantitative PCR of stemness-related genes in HLE (**left**) and HLF (**right**) cells treated with DMSO and BRD4354 (5 and 10 μM). Quantitative PCR analysis was performed at *n* = 4. * *p* < 0.05, ** *p* < 0.01 vs. DMSO group. (**c**) Cell proliferation assays of HLE (**left**) and HLF (**right**) cells treated with 0, 0.2, 0.5, and 1.0 μM of disulfiram (DSF) for 3 days. Cell viability was determined by WST assay and expressed as the relative amount of viable cells compared to day 0. (**d**) Spheres assay of HLE and HLF cells treated with 0, 0.2, and 0.5 μM of DSF for 7 days. The diameter of the sphere was measured in five representative spheres of each group. * *p* < 0.05, ** *p* < 0.01 vs. vehicle control.

To find the molecular link between HDAC9 expression and anchorage-independent cell growth in undifferentiated HCC, we investigated the gene expression of stemness-related genes in si-HDAC9-treated cells. Among the genes examined, ALDH1A3 was significantly decreased in the si-HDAC9-transduced HLE cells (Figure 5a). On the other hand, ALDH1A3 was barely expressed in HLF cells and the other stemness genes, including Oct4, Bmi1, CD44, and ALDH1A1, were not changed by HDAC9 knockdown. ALDH1A1, a major isoform of ALDH was strongly expressed in both HLE and HLF cells, however, showed little alteration of gene expression by si-RNA-mediated HDAC9 knockdown in both cell lines. Interestingly, pharmacological inhibition by BRD4354 down-regulated more stemness genes compared to si-HDAC9 knockdown in both cell lines (Figure 5b). Next, we examined the effect of ALDH inhibition on anchorage-dependent and independent cell growth by using the pan-ALDH inhibitor disulfiram. Cell proliferation in the monolayer culture was not affected by the treatment of 0.2–1.0 μM disulfiram in both cell lines (Figure 5c). In contrast, these concentrations of disulfiram significantly decreased the anchorage-independent growth in the sphere assay (Figure 5d). These results provide a possibility that ALDH activity is involved in the acquisition of stemness features in HDAC9-expressing undifferentiated HCC cells.

## 3. Discussion

Dedifferentiation of cancer cells is a crucial cue for malignant transformation and progression. To improve the prognosis of patients, it is important to identify the factors that regulate the dedifferentiation process in order to develop target-specific drugs and gene therapies. In this study, we aimed to identify the HDAC that is responsible for the HCC cell dedifferentiation and found HDAC9 as a target molecule for inhibiting stemness features. HDAC9 is identified as a member of the class II HDACs whose N-terminal domains have an MEF-2 interacting region [12]. HDAC9 mRNA and its alternative spliced isoform HDRP are reported to be expressed throughout various tissues while its expression is relatively low in liver [12,13]. We observed a minimum expression of HDAC9 in the normal hepatic cell line HC and well-differentiated HepG2 and HuH1 hepatoma cells that retain a hepatic phenotype, such as hepatic gene expression and drug-metabolizing activities. In contrast, HDAC9 mRNA was positively detected in HLE and HLF cells, typical undifferentiated HCC cell lines that show a mesenchymal phenotype [14,15,16]. Consistent with previous reports, class I HDACs were ubiquitously expressed among the cell lines tested, suggesting that HDAC9, but not class I HDACs, may play a specific role in undifferentiated HCC cells [17]. Analysis of the TCGA database revealed that HDAC9 mRNA expression in human HCC patients correlated positively with the expression of marker genes of mesenchymal phenotype and stemness, while correlating negatively with those of hepatic marker genes. A recent study reports that HDAC9 expression is associated with poor prognosis, being an independent prognostic parameter in a study cohort with 37 HCC patients [18]. These reports and our results provide a hypothesis that HDAC9 is involved in the dedifferentiation process of HCC cells.

EMT is one of the dedifferentiation processes of epithelial tumor cells toward a mesenchymal phenotype, and is a major obstacle for effective cancer treatment. TGF-β-induced EMT has been established as a model of EMT in many cancers [19]. We observed typical morphological changes from epithelial to mesenchymal cell shape and expression change of marker genes in TGF-β-stimulated HuH1 cells. HDAC genes showed differential regulation in that only the HDAC9 gene was up-regulated, and class I HDACs were down-regulated during the EMT process. A number of studies have shown an involvement of HDAC9 in the ability of migration and invasion [20,21,22,23]. However, in the case of TGF-β-induced EMT, HDAC9 suppression barely inhibited the phenotypic change of HuH1 cells, suggesting that HDAC9 does not locate in the upstream of EMT induction mediated by TGF-β-SMAD or non-SMAD pathway [19]. HDAC9 inhibition by si-RNA and chemical inhibitor did not reduce the motility of undifferentiated HCC cells. Therefore, upregulation of HDAC9 may be one of the phenotypic changes of EMT that is upregulated in the mesenchymal type of cells. In endothelial cells, HDAC9 is induced via signal transducer and activator of transcription 3 (STAT3) signaling to proliferate by PDAC-secreted proangiogenic factors [24]. HDAC9 is reported to be regulated post-transcriptionally by several micro RNAs in retinal, oral, breast, and gastric cancer, and as being associated with poor prognosis [21,23,25,26]. Analysis of other signaling pathways that induce dedifferentiation and malignant phenotype via HDAC9 may help us to understand the essential role of HDAC9 in tumor dedifferentiation.

Anchorage-independent cell growth and self-renewal ability are the important features of cancer stem cells (CSCs) to drive tumorigenesis after metastasis. CSCs are a sub-population of tumor cells responsible for their initiation, recurrence, and metastasis. Previously, we observed a 0.1–1.0% sphere formation rate in HLE and HLF cells seeded in a low-attachment dish [16]. In this study, HDAC9 suppression significantly reduced the growth of these spheres in both cell lines. Quantitative PCR revealed that sphere-forming cells expressed much higher HDAC9 expression than 2D-cultured population. These results suggest that HDAC9 plays a crucial role in the sphere-formation of undifferentiated HCC cells. Similar results were reported in retinoblastoma and gastric cancer, however, the molecular pathway which links HDAC9 to the stemness property remains unknown [21,27].

Considering the question above, we searched the stemness-related genes whose expression or activity may be affected by HDAC9. Gene expression analysis of HDAC9-suppressed cells showed significant down-regulation of the ALDH1A3 gene in HLE cells. ALDH1A3 was a gene of positive correlation with HDAC9 in TCGA analysis (Table 1). Accumulating evidence indicates that ALDH activity could be used for identifying a subpopulation of CSCs in many types of cancer including HCC [28,29]. We therefore performed sphere-forming assays in the presence of the ALDH inhibitor disulfiram and confirmed that sphere-forming activity was significantly affected by the ALDH inhibitor at the low concentrations that have no inhibitory effect in 2D proliferation. This observation suggests that ALDH activity is the important regulator of stemness in undifferentiated HCC cells. It is reported that ALDH1A3 has higher catalytic efficiency than ALDH1A1 [30]. The ALDH1A3 isotype has been reported to be responsible for ALDH activity in cardiomyocyte, breast cancer, and cholangiocarcinoma cells [31,32,33,34]. These reports support the potential role of ALDH1A3 in conferring stemness properties in undifferentiated HCC cells. Interestingly, BRD4354 was able to down-regulate more stemness genes than si-HDAC9 knockdown in undifferentiated HCC cells (Figure 5b). This result may be partly explained by the difference of target specificity of inhibition, with BRD4354 reported to inhibit the other HDACs, including class I and class II, to a lesser degree [35].

Although ALDH1A1 is abundantly expressed in HLE and HLF cells, HDAC9 suppression barely affected the transcript level of ALDH1A1 in both cells. In the literature, ALDH1A1 is post-transcriptionally regulated by SIRT-mediated deacetylation at Lys353 in lung cancer cells [36,37]. In addition, the HDAC9 variant that showed cytoplasmic localization due to the lack of nuclear localizing signal (NLS) sequence was identified [38]. Class II HDACs, including HDAC5, HDAC6, and HDAC9, have been reported to catalyze the deacetylation of cytoplasmic, non-histone proteins in cancer cells [39,40,41]. These reports allowed us to speculate that ALDH1A1 might be activated through a deacetylation by cytoplasmic HDAC9 variant. Although we only demonstrated the relationship between HDAC9 and ALDHs at the mRNA level, further study, including protein expression, acetylation status, and enzymatic activity of ALDH, will unveil the molecular mechanism of dedifferentiation regulated by HDAC9.

**Figure 6:**
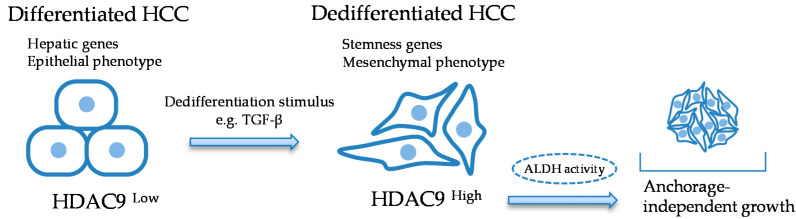
Schematic view of the study. HDAC9 is preferentially expressed in dedifferentiated HCC cells and is involved in an anchorage-independent growth. A broken line indicates the hypothesis needing to be clarified in the future study.

In summary, we found that the class II HDAC9 is a regulator of the differentiation and acquisition of stemness properties in HCC cells. TCGA analysis and the in vitro experiments suggest that the ability of anchorage-independent growth maintained by HDAC9 may be partly due to the regulation of ALDH (Figure 6). Our findings provide useful information for drug development and gene therapies that target specific HDAC for cancer treatment.

## 4. Materials and Methods

### 4.1. Cell Culture and Reagents

Hepatoma cell lines (HepG2, HuH1, HLE and HLF) were purchased from Riken BioResource Research Center (RIKEN BRC). The human hepatic cell line Hc was kindly provided by Dr. Qin X.Y. of RIKEN, Japan. Cells were maintained in Dulbecco’s Modified Eagle Medium (DMEM) containing 10% FBS, 4.5 mg/mL glucose, and 2 mM L-glutamine in a humidified incubator at 37 °C and 5% CO_2_. TGF-β (Peprotech; Cranbury, NJ, USA), BRD4354 (Tocris Biosciences, Minneapolis, MN, USA), and disulfiram (Tokyo Chemical Industry Co., Ltd., Tokyo, Japan) were dissolved in a vehicle and stored in a freezer at −20 °C.

### 4.2. Cell Proliferation Assays

For cell proliferation assays, cells were seeded at 3 × 10^3^ cells/well in a 96-well plate and subjected to the experiment. Cell viability was determined by the water-soluble tetrazolium (WST) assay using the Cell Counting Kit-8 (Dojin Chemical Co., Ltd.; Kumamoto, Japan) at 24, 48, and 72 h after the treatment. To determine cell viability, a 10% volume of CCK-8 reagent was added to each well, and the plate was incubated for 1 h at 37 °C. The relative amount of viable cells was calculated by measuring the absorbance at a wavelength of 450 nm.

### 4.3. Gene Expression Analysis

Total RNA was extracted from the cells by using the TRIZOL reagent in accordance with the manufacturer’s instructions (Invitrogen; Carlsbad, CA, USA). Half of a microgram of RNA was used to synthesize cDNA using GeneAce Reverse Transcriptase (Nippon Gene; Tokyo, Japan). A gene expression analysis was performed using PowerUp SYBR Green Master Mix (Thermo Fisher Scientific; Waltham, MA USA) and the Applied Biosystems (ABI) qPCR system (Thermo Fisher Scientific). The primers used in this study are listed in Table S1. In public database analysis, the dataset of Liver Hepatocellular Carcinoma (Firehose Legacy) in The Cancer Genome Atlas (TCGA) database was analyzed by using the cBioPortal for Cancer Genomics (https://www.cbioportal.org/). Samples with mRNA expression data (*n* = 373) were included for the screening of co-expression genes.

### 4.4. Western Blot Analysis

Cell lysates were prepared from the cells subjected to the experiment by using Cell Lysis Buffer (Cell Signaling Technology; Danvers, MA, USA) and stored in a deep freezer until use. The protein concentration was determined by the Pierce™ BCA Protein Assay Kit (Thermo Fisher Scientific). Equal amounts of the protein sample were separated on a SDS-PAGE and immunoblotted with antibodies against human HDAC9 (Santa Cruz Biotechnology, Dallas, TX, USA), E-cadherin (Santa Cruz Biotechnology), vimentin (Santa Cruz Biotechnology), and beta-actin (Cell Signaling Technology). Horseradish peroxidase (HRP)-conjugated secondary antibodies were used to detect primary antibodies and were visualized with ECL detection reagent (GE Healthcare UK; Buckinghamshire, UK) and a light-sensitive X-ray film (Fuji film; Tokyo, Japan). The band images were analyzed by Image J software (NIH, Bethesda, MD, USA) for a quantification of protein expression. Uncropped western blot figures for Figure 1b, Figure 2b, and Figure 2f are shown in Figure S4.

### 4.5. Small-Interference RNA Experiment

A gene-knockdown experiment was performed by transfection of Stealth RNAi Pre-Designed siRNAs (Thermo Fisher Scientific) for HDAC9 mRNA (si-HDAC9) and a negative control (si-Control). Briefly, siRNA and Lipofectamine3000 (Thermo Fisher Scientific) were dissolved in Opti-MEM^®^ I Reduced Serum Media (Thermo Fisher Scientific) separately, and then mixed to form transfection complex for 15 min. A final concentration of 10 pmol/mL of siRNA was used for the knockdown experiments.

### 4.6. Sphere-Forming Assay

A sphere assay was performed by culturing HLE and HLF cells in a 24-well low-attachment plate (Sumitomo Bakelite Co., Ltd.; Tokyo, Japan). Briefly, cells were seeded at 500 cells/well and treated with the reagent used in the experiments. Half of the medium was changed every three days. After 8 days of culture, formed spheres were photographed and their diameters were measured.

### 4.7. Statistical Analysis

Statistical comparisons for in vitro experiments were performed using Student’s *t*-tests. *p* < 0.05 was considered statistically significant.

## 5. Conclusions

We identified HDAC9 as a key regulator of cell growth and stemness in undifferentiated HCC cells. Inhibition of HDAC9 may be a therapeutic strategy for targeting dedifferentiated HCC cells with stemness features.

## Figures and Tables

**Table 1:** List of genes correlated with HDAC9 expression in 373 HCC samples in the The Cancer Genome Atlas (TCGA) database.

Correlated Gene	Positive or Negative	Spearman’s Correlation	*p*-Value ^1^
Mesenchymal markers			
VIM	positive	0.616	2.35 × 10^−40^
SNAI1	positive	0.473	3.82 × 10^−22^
TWIST1	positive	0.452	3.42 × 10^−20^
ZEB1	positive	0.318	3.21 × 10^−10^
ZEB2	positive	0.727	1.34 × 10^−62^
Stemness markers			
CD44	positive	0.417	4.03 × 10^−17^
ALDH1L2	positive	0.624	1.42 × 10^−41^
ALDH1A3	positive	0.424	1.04 × 10^−17^
KRT19	positive	0.411	1.14 × 10^−16^
Proliferation marker			
CCND2	positive	0.634	2.41 × 10^−43^
Hepatic markers			
HNF1A	negative	−0.421	1.93 × 10^−17^
HNF4A	negative	−0.299	3.88 × 10^−9^
FOXA2	negative	−0.38	2.84 × 10^−14^

^1^*p*-Values were derived from 2-sided *t*-test.

## Supplementary Materials

The following are available online at https://www.mdpi.com/2072-6694/12/10/2734/s1, Figure S1: Wound healing cell migration assay of control and HDAC 9 suppressed cells, Figure S2: Drug sensitivity of control and HDAC 9 suppressed undifferentiated HCC cells, Figure S3: Wound healing cell migration assay of the cells treated with HDAC 9 inhibitor, Figure S4: Uncropped western blot figures, Table S1: Primers used in this study.

## References

[1] Csordas A. (1990). On the biological role of histone acetylation. Biochem. J..

[2] Barneda-Zahonero B., Parra M. (2012). Histone deacetylases and cancer. Mol. Oncol..

[3] Subramanian S., Bates S.E., Wright J.J., Espinoza-Delgado I., Piekarz R.L. (2010). Clinical toxicities of histone deacetylase Inhibitors. Pharmaceuticals.

[4] Yang J.D., Hainaut P., Gores G.J., Amadou A., Plymoth A., Roberts L.R. (2019). A global view of hepatocellular carcinoma: Trends, risk, prevention and management. Nat. Rev. Gastroenterol. Hepatol..

[5] Gupta P.B., Pastushenko I., Skibinski A., Blanpain C., Kuperwasser C. (2019). Phenotypic Plasticity: Driver of Cancer Initiation, Progression, and Therapy Resistance. Cell Stem Cell.

[6] Flores-Téllez T.N., Villa-Treviño S., Piña-Vázquez C. (2017). Road to stemness in hepatocellular carcinoma. World J. Gastroenterol..

[7] Lee Y.H., Seo D., Choi K.J., Andersen J.B., Won M.A., Kitade M., Gómez-Quiroz L.E., Judge A.D., Marquardt J.U., Raggi C. (2014). Antitumor effects in hepatocarcinoma of isoform-selective inhibition of HDAC2. Cancer Res..

[8] Ler S.Y., Leung C.H., Khin L.W., Lu G.D., Salto-Tellez M., Hartman M., Iau P.T., Yap C.T., Hooi S.C. (2015). HDAC1 and HDAC2 independently predict mortality in hepatocellular carcinoma by a competing risk regression model in a Southeast Asian population. Oncol. Rep..

[9] Tsai C.L., Liu W.L., Hsu F.M., Yang P.S., Yen R.F., Tzen K.Y., Cheng A.L., Chen P.J., Cheng J.C. (2018). Targeting histone deacetylase 4/ubiquitin-conjugating enzyme 9 impairs DNA repair for radiosensitization of hepatocellular carcinoma cells in mice. Hepatology.

[10] Zhang M., Pan Y., Dorfman R.G., Chen Z., Liu F., Zhou Q., Huang S., Zhang J., Yang D., Liu J. (2016). AR-42 induces apoptosis in human hepatocellular carcinoma cells via HDAC5 inhibition. Oncotarget.

[11] Kanki K., Watanabe R., Le N.T., Zhao C.H., Naito K. (2019). Personal communication/observation.

[12] Zhou X., Marks P.A., Rifkind R.A., Richon V.M. (2001). Cloning and characterization of a histone deacetylase, HDAC9. Proc. Natl. Acad. Sci. USA.

[13] Zhou X., Richon V.M., Rifkind R.A., Marks P.A. (2000). Identification of a transcriptional repressor related to the noncatalytic domain of histone deacetylases 4 and 5. Proc. Natl. Acad. Sci. USA.

[14] Okabe H., Ishimoto T., Mima K., Nakagawa S., Hayashi H., Kuroki H., Imai K., Nitta H., Saito S., Hashimoto D. (2014). CD44s signals the acquisition of the mesenchymal phenotype required for anchorage-independent cell survival in hepatocellular carcinoma. Br. J. Cancer.

[15] Mima K., Okabe H., Ishimoto T., Hayashi H., Nakagawa S., Kuroki H., Watanabe M., Beppu T., Tamada M., Nagano O. (2012). CD44s regulates the TGF-beta-mediated mesenchymal phenotype and is associated with poor prognosis in patients with hepatocellular carcinoma. Cancer Res..

[16] Harada K., Ohashi R., Naito K., Kanki K. (2020). Hedgehog Signal Inhibitor GANT61 Inhibits the Malignant Behavior of Undifferentiated Hepatocellular Carcinoma Cells by Targeting Non-Canonical GLI Signaling. Int. J. Mol. Sci..

[17] Freese K., Seitz T., Dietrich P., Lee S.M.L., Thasler W.E., Bosserhoff A., Hellerbrand C. (2019). Histone Deacetylase Expressions in Hepatocellular Carcinoma and Functional Effects of Histone Deacetylase Inhibitors on Liver Cancer Cells In Vitro. Cancers.

[18] Hu Y., Sun L., Tao S., Dai M., Wang Y., Li Y., Wu J. (2019). Clinical significance of HDAC9 in hepatocellular carcinoma. Cell. Mol. Biol..

[19] Hao Y., Baker D., Ten Dijke P. (2019). TGF-β-Mediated Epithelial-Mesenchymal Transition and Cancer Metastasis. Int. J. Mol. Sci..

[20] Li H., Li X., Lin H., Gong J. (2020). High HDAC9 is associated with poor prognosis and promotes malignant progression in pancreatic ductal adenocarcinoma. Mol. Med. Rep..

[21] Xu L., Li W., Shi Q., Wang M., Li H., Yang X., Zhang J. (2020). MicroRNA-936 inhibits the malignant phenotype of retinoblastoma by directly targeting HDAC9 and deactivating the PI3K/AKT pathway. Oncol. Rep..

[22] Huang Y., Jian W., Zhao J., Wang G. (2018). Overexpression of HDAC9 is associated with poor prognosis and tumor progression of breast cancer in Chinese females. Onco Targets Ther..

[23] Rastogi B., Kumar A., Raut S.K., Panda N.K., Rattan V., Joshi N., Khullar M. (2017). Downregulation of miR-377 Promotes Oral Squamous Cell Carcinoma Growth and Migration by Targeting HDAC9. Cancer Investig..

[24] Gore J., Craven K.E., Wilson J.L., Cote G.A., Cheng M., Nguyen H.V., Cramer H.M., Sherman S., Korc M. (2015). TCGA data and patient-derived orthotopic xenografts highlight pancreatic cancer-associated angiogenesis. Oncotarget.

[25] Liang Z., Feng A., Shim H. (2020). MicroRNA-30c-regulated HDAC9 mediates chemoresistance of breast cancer. Cancer Chemother. Pharmacol..

[26] Xu G., Li N., Zhang Y., Zhang J., Xu R., Wu Y. (2019). MicroRNA-383-5p inhibits the progression of gastric carcinoma via targeting HDAC9 expression. Braz. J. Med. Biol. Res..

[27] Xiong K., Zhang H., Du Y., Tian J., Ding S. (2019). Identification of HDAC9 as a viable therapeutic target for the treatment of gastric cancer. Exp. Mol. Med..

[28] Rodriguez-Torres M., Allan A.L. (2016). Aldehyde dehydrogenase as a marker and functional mediator of metastasis in solid tumors. Clin. Exp. Metastasis.

[29] Ma S., Chan K.W., Lee T.K., Tang K.H., Wo J.Y., Zheng B.J., Guan X.Y. (2008). Aldehyde dehydrogenase discriminates the CD133 liver cancer stem cell populations. Mol. Cancer Res..

[30] Pequerul R., Vera J., Giménez-Dejoz J., Crespo I., Coines J., Porté S., Rovira C., Parés X., Farrés J. (2020). Structural and kinetic features of aldehyde dehydrogenase 1A (ALDH1A) subfamily members, cancer stem cell markers active in retinoic acid biosynthesis. Arch. Biochem. Biophys..

[31] Puttini S., Plaisance I., Barile L., Cervio E., Milano G., Marcato P., Pedrazzini T., Vassalli G. (2018). ALDH1A3 Is the Key Isoform That Contributes to Aldehyde Dehydrogenase Activity and Affects in Vitro Proliferation in Cardiac Atrial Appendage Progenitor Cells. Front. Cardiovasc. Med..

[32] Croker A.K., Rodriguez-Torres M., Xia Y., Pardhan S., Leong H.S., Lewis J.D., Allan A.L. (2017). Differential Functional Roles of ALDH1A1 and ALDH1A3 in Mediating Metastatic Behavior and Therapy Resistance of Human Breast Cancer Cells. Int. J. Mol. Sci..

[33] Marcato P., Dean C.A., Pan D., Araslanova R., Gillis M., Joshi M., Helyer L., Pan L., Leidal A., Gujar S. (2011). Aldehyde dehydrogenase activity of breast cancer stem cells is primarily due to isoform ALDH1A3 and its expression is predictive of metastasis. Stem Cells.

[34] Chen M.H., Weng J.J., Cheng C.T., Wu R.C., Huang S.C., Wu C.E., Chung Y.H., Liu C.Y., Chang M.H., Chen M.H. (2016). ALDH1A3, the Major Aldehyde Dehydrogenase Isoform in Human Cholangiocarcinoma Cells, Affects Prognosis and Gemcitabine Resistance in Cholangiocarcinoma Patients. Clin. Cancer Res..

[35] Boskovic Z.V., Kemp M.M., Freedy A.M., Viswanathan V.S., Pop M.S., Fuller J.H., Martinez N.M., Figueroa, Lazú S.O., Hong J.A., Lewis T.A. (2016). Inhibition of Zinc-Dependent Histone Deacetylases with a Chemically Triggered Electrophile. ACS Chem. Biol..

[36] Zhao D., Mo Y., Li M.T., Zou S.W., Cheng Z.L., Sun Y.P., Xiong Y., Guan K.L., Lei Q.Y. (2014). NOTCH-induced aldehyde dehydrogenase 1A1 deacetylation promotes breast cancer stem cells. J. Clin. Investig..

[37] Sullivan J.P., Spinola M., Dodge M., Raso M.G., Behrens C., Gao B., Schuster K., Shao C., Larsen J.E., Sullivan L.A. (2010). Aldehyde dehydrogenase activity selects for lung adenocarcinoma stem cells dependent on notch signaling. Cancer Res..

[38] Petrie K., Guidez F., Howell L., Healy L., Waxman S., Greaves M., Zelent A. (2003). The histone deacetylase 9 gene encodes multiple protein isoforms. J. Biol. Chem..

[39] Azuma K., Urano T., Horie-Inoue K., Hayashi S., Sakai R., Ouchi Y., Inoue S. (2009). Association of estrogen receptor alpha and histone deacetylase 6 causes rapid deacetylation of tubulin in breast cancer cells. Cancer Res..

[40] Yuan Z., Peng L., Radhakrishnan R., Seto E. (2010). Histone deacetylase 9 (HDAC9) regulates the functions of the ATDC (TRIM29) protein. J. Biol. Chem..

[41] Chen S., Yin C., Lao T., Liang D., He D., Wang C., Sang N. (2015). AMPK-HDAC5 pathway facilitates nuclear accumulation of HIF-1α and functional activation of HIF-1 by deacetylating Hsp70 in the cytosol. Cell Cycle.

