# Supplementary material for: HDAC9 Is Preferentially Expressed in Dedifferentiated Hepatocellular Carcinoma Cells and Is Involved in an Anchorage-Independent Growth
Source: Cancers (Basel). 2020 Sep 23;12(10):2734. doi: 10.3390/cancers12102734 (PMC7598174; doi:10.3390/cancers12102734)
Supplement: Supplementary file 1 [file cancers-12-02734-s001.pdf]

Supplementary Materials: HDAC9 Is Preferentially Expressed in Dedifferentiated Hepatocellular Carcinoma Cells and Is Involved in an Anchorage-Independent Growth

Keita Kanki, Ryota Watanabe, Le Nguyen Thai, Chun-Hao Zhao and Kyoko Naito

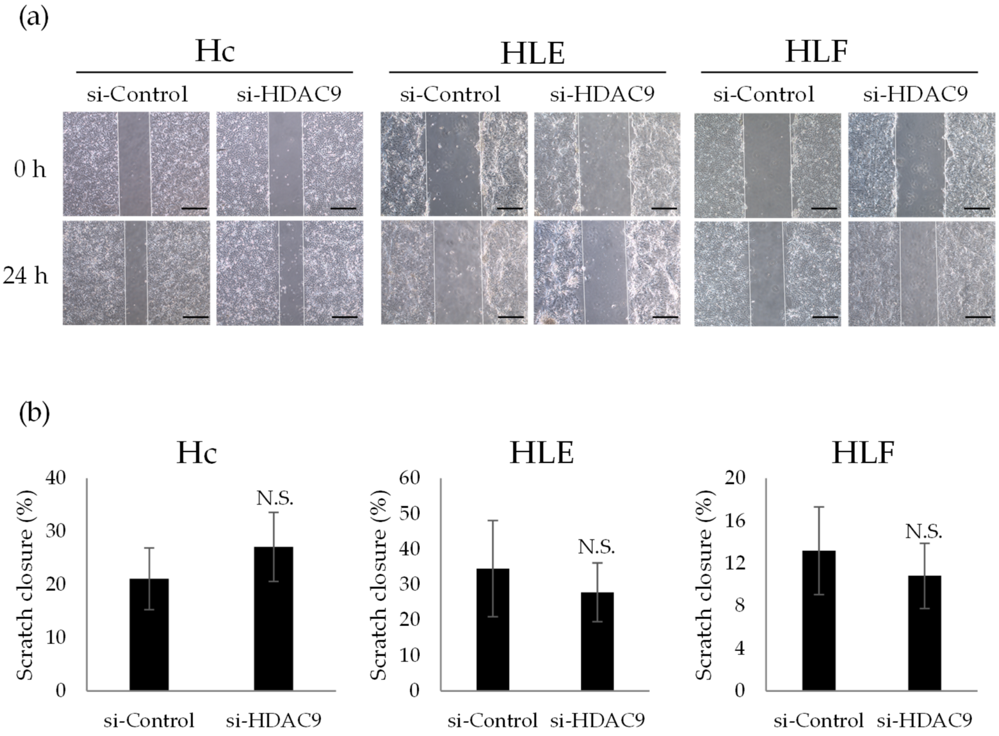

Figure S1. Wound-healing cell migration assay of control and HDAC9-suppressed cells. Monolayer-cultured cells were transduced with si-control and si-HDAC9 for 24 h, and then, were scratched with microtip and photographed (0 h). Next day, the scratches were photographed (24 h) and the same scratch was measured to evaluated the cell migration activity. (**a**) Representative images of scratches photographed at 0 h (upper) and 24 h (lower) in Hc (left), HLE (center), and HLF (right) cells. Bar = 250 μm (**b**) Cell migration activity determined by measuring scratch closure for 24 h. n = 10, N.S.; not significant vs. si-control.

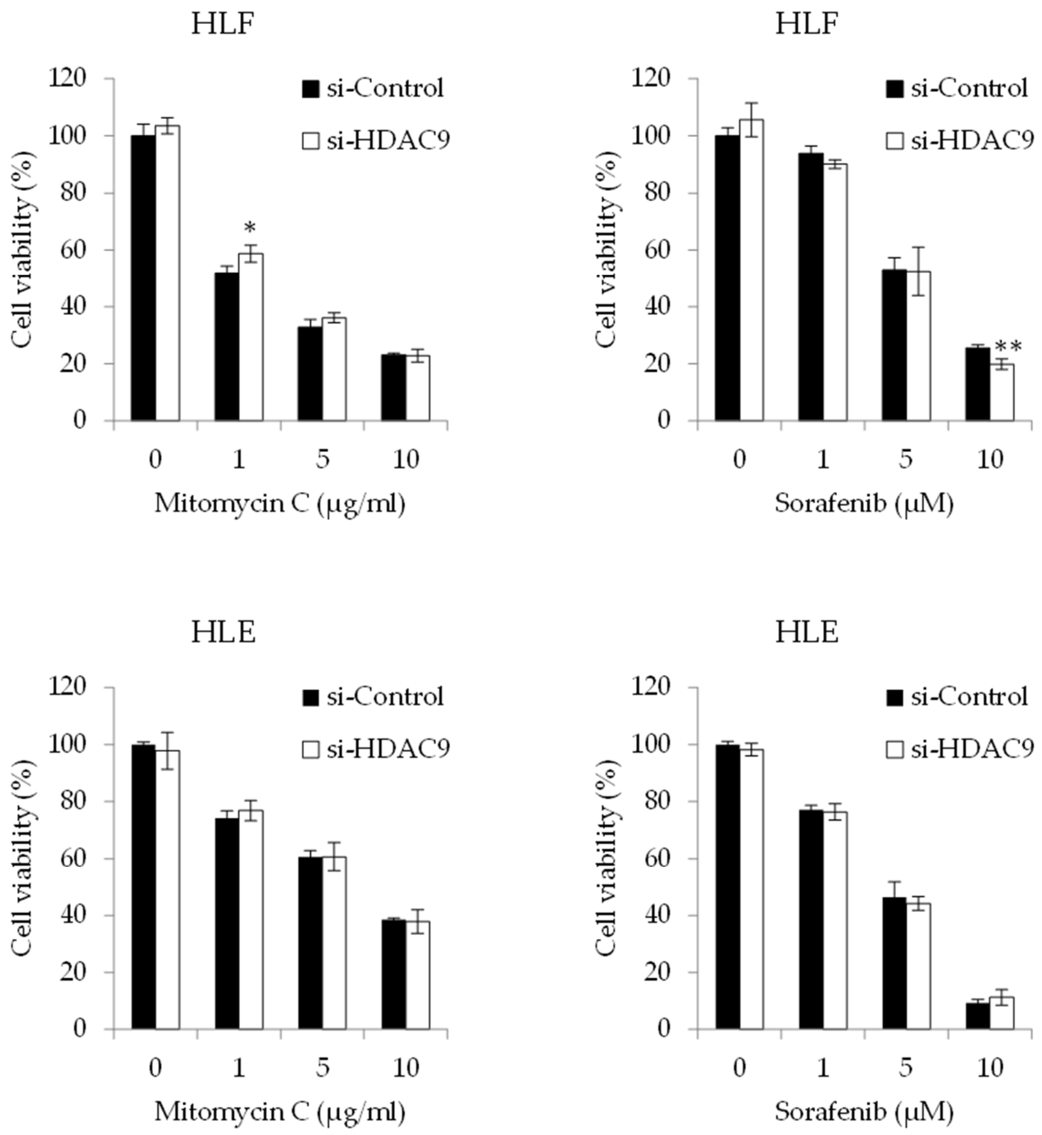

Figure S2. Drug sensitivity of control and HDAC9-suppressed undifferentiated HCC cells. Cells were transduced with si-control (closed bar) and si-HDAC9 (open bar) for 24 h, and then, were treated with mitomycin C (0–10 μg/mL) and sorafenib (0–10 μM) for 48 h. Cell viability was measured by Cell Counting Kit-8 and is expressed as the percentage of drug-free control. n = 3, *p < 0.05, **p < 0.01 vs. si-control.

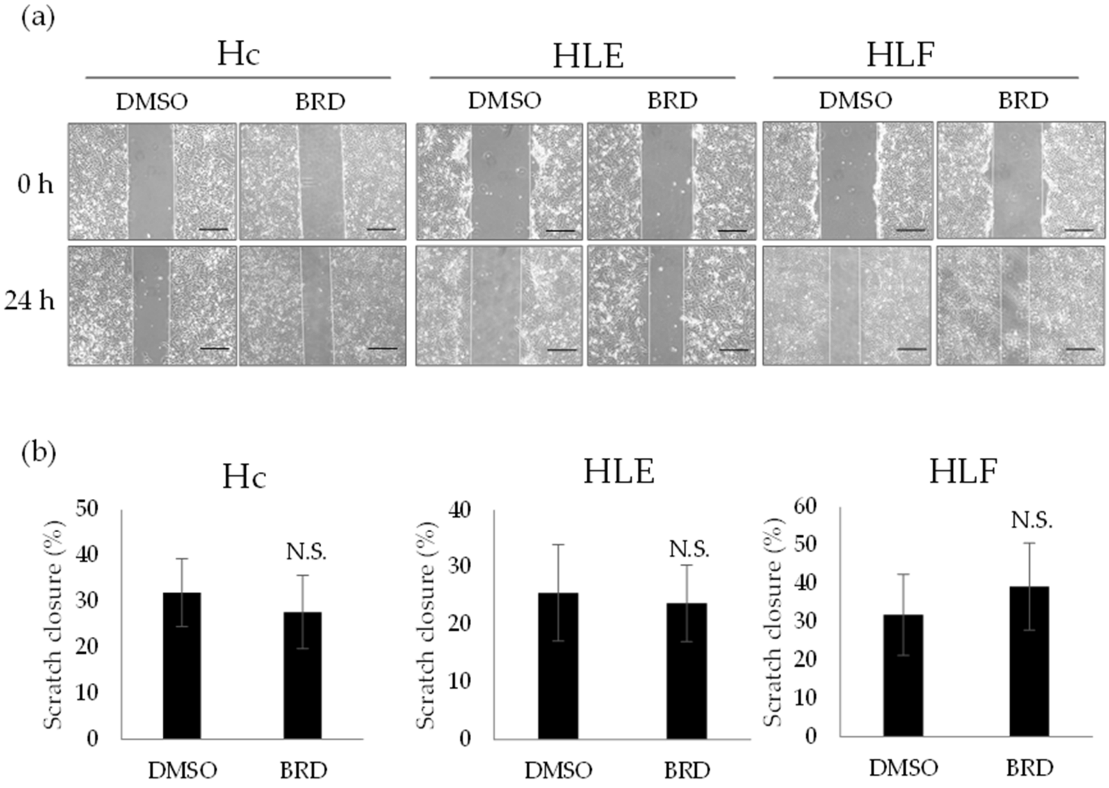

Figure S3. Wound-healing cell migration assay of the cells treated with HDAC9 inhibitor. Monolayer-cultured cells were treated with DMSO (control) and BRD4354 (5 μM) for 24 h, and then, were scratched with microtip and photographed (0 h). Next day, the scratches were photographed (24 h) and the same scratch was measured to evaluated the cell migration activity. (**a**) Representative images of scratches photographed at 0 h (upper) and 24 h (lower) in Hc (left), HLE (center), and HLF (right) cells. Bar = 250 μm (**b**) Cell migration activity determined by measuring scratch closure for 24 h. n = 10, N.S.; not significant vs. DMSO.

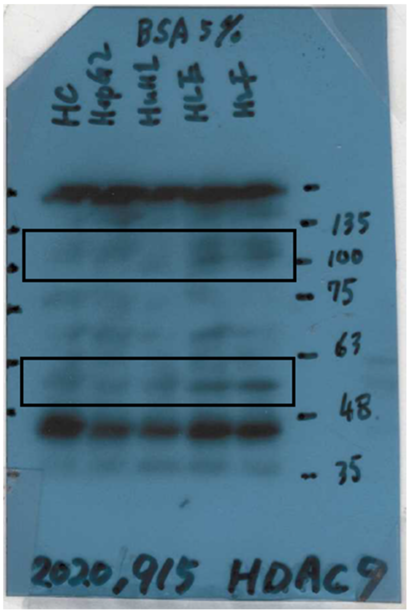

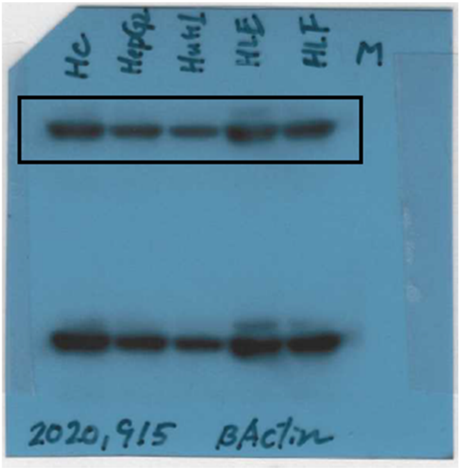

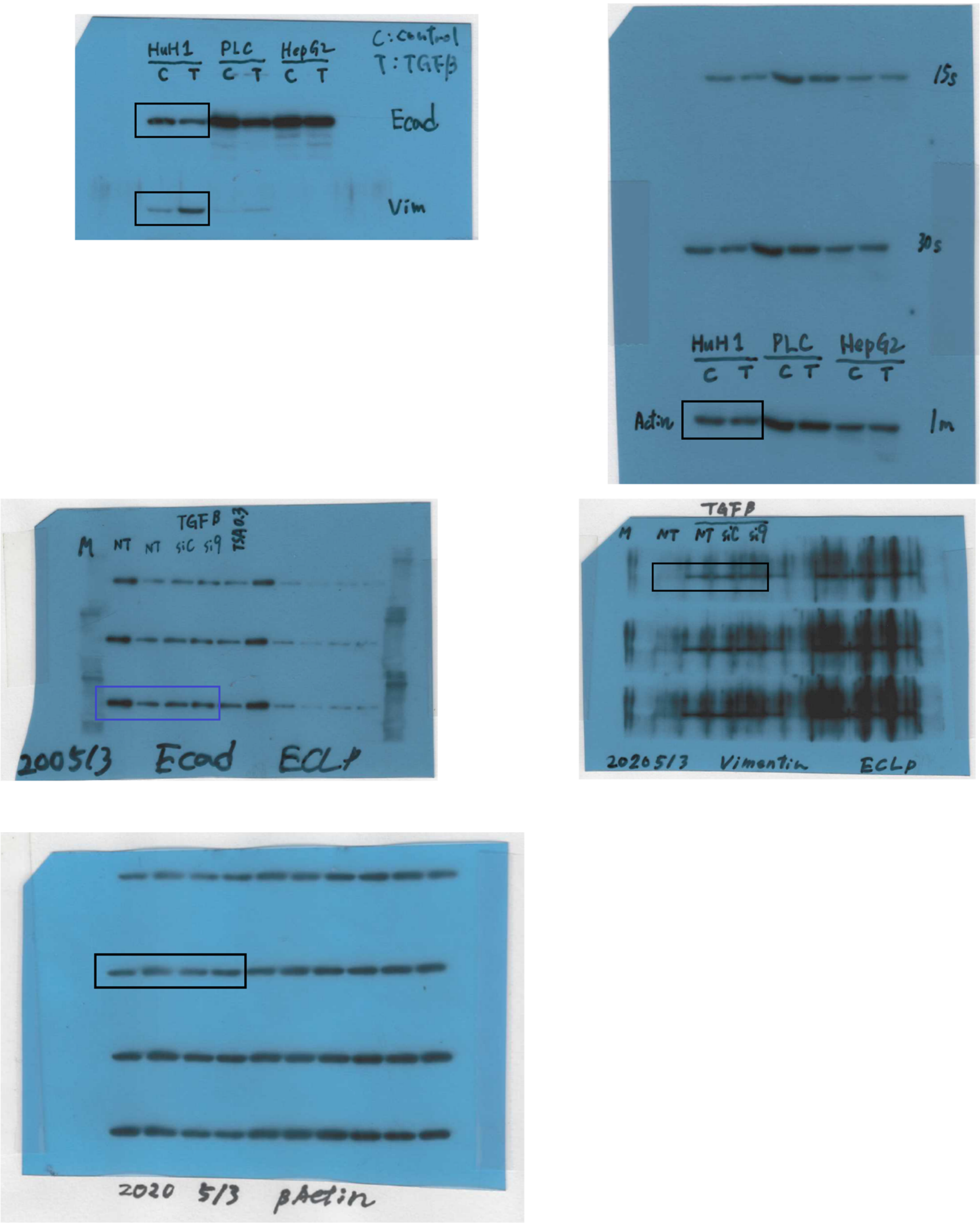

Figure S4. Uncropped western blot figures. Figure. 1b, Figure. 2b, Figure. 2f. Protein-transferred membranes were detected by using anti-HDAC9, anti-E-cadherin, anti-vimentin, and anti-β-actin. Horseradish peroxidase (HRP)-conjugated secondary antibodies were used to detect primary antibodies and were visualized with chemiluminescence-based detection reagent and a light-sensitive X-ray film. The molecular weight deduced from a molecular marker lane (M) was indicated.

Table S1. Primers used in this study.

Gene	Sense	Anti-sense
HDAC1	GAGACGGGATTGATGACGAG	CCACAGCTGTCTCATATGTCC
HDAC2	ACCGACAACAGACTGATATGGC	CTCAAGTCTCCTGTGCCAGG
HDAC3	GACCTATGACAGGACTGATGAGG	CAGACTCTTTCCCAGAGTCAGC
HDAC8	GTCCCGGTTTATATCTATAGTCCC	CTTCTGGAGATGCTGCAGATAAG
HDAC9	GGAGCAGAAACTGGAGCAG	CAATGATGTGTGGTGGGC
E-cadherin	CTGGCCAAGGAGCTGACAC	CTTGTTGTCATTCTGATCGGTT
AFP	CTGCAATTGAGAAACCCACTG	ACAGCTTGTGACAGGTTCTGG
Albumin	GACCTTGCCAAGTATATCTG	CAGGAAGACATCCTTTGC
CK7	ACAGAGCTGCAGTCCCAGA	GATCTCAGCCTGCAGCCTC
CK19	AGATCCGCGACTGGTACC	ATCTCCAGGTCGGTCCTG
Oct4	ATTCAGCCAAACGACCAT	GGTTCGCTTTCTCTTTCGG
Bmi1	AAGATACTTACGATGCCCAGC	TCCAGTTCTCCAGCATTTGTC
ALDH1A1	AAGAGATCGTCTGCTGCTGG	CAATTGGTATTGTACGGCCC
ALDH1A3	TCCCGGAGCAATCTGAAG	TCCGCCTGACAAACTCAGAG
β-actin	CACTCTTCCAGCCTTCCTTCC	CGTACAGGTCTTTGCGGATGTC

© 2020 by the authors. Submitted for possible open access publication under the terms and conditions of the Creative Commons Attribution (CC BY) license (http://creativecommons.org/licenses/by/4.0/).
